# Low Percentage of Vegetable Fat in Red Blood Cells Is Associated with Worse Glucose Metabolism and Incidence of Type 2 Diabetes

**DOI:** 10.3390/nu14071368

**Published:** 2022-03-25

**Authors:** Gemma Chiva-Blanch, Oriol Giró, Montserrat Cofán, Alfonso L. Calle-Pascual, Elías Delgado, Ramon Gomis, Amanda Jiménez, Josep Franch-Nadal, Gemma Rojo Martínez, Emilio Ortega

**Affiliations:** 1 Department of Endocrinology and Nutrition, August Pi i Sunyer Biomedical Research Institute-IDIBAPS, Hospital Clínic of Barcelona, 08036 Barcelona, Spain; gchiva@clinic.cat (G.C.-B.); giroi@clinic.cat (O.G.); mcofan@clinic.cat (M.C.); ramon.gomis@idibaps.org (R.G.); ajimene1@clinic.cat (A.J.); 2 Spanish Biomedical Research Network in Physiopathology of Obesity and Nutrition (CIBEROBN), Instituto de Salud Carlos III, 28029 Madrid, Spain; 3 Spanish Biomedical Research Network in Diabetes and Associated Metabolic Disorders (CIBERDEM), Instituto de Salud Carlos III, 28029 Madrid, Spain; acalle.edu@gmail.com (A.L.C.-P.); josep.franch@gmail.com (J.F.-N.); gemma.rojo.m@gmail.com (G.R.M.); 4 Department of Endocrinology and Nutrition, San Carlos University Hospital of Madrid, 28040 Madrid, Spain; 5 Spanish Biomedical Research Network in Rare Diseases (CIBERER), Instituto de Salud Carlos III, 28029 Madrid, Spain; delgadoelias@uniovi.es; 6 Department of Endocrinology and Nutrition, Central University Hospital of Asturias, University of Oviedo, Health Research Institute of the Principality of Asturias (ISPA), 33011 Oviedo, Spain; 7 EAP Raval Sud, Catalan Institute of Health, GEDAPS Network, Primary Care, Research Support Unit (IDIAP-Jordi Gol Foundation), 08001 Barcelona, Spain; 8 Biomedical Research Institute of Malaga (IBIMA), Endocrinology and Nutrition Department, Regional University Hospital of Malaga, 29010 Malaga, Spain

**Keywords:** type 2 diabetes, worse of glucose metabolism, fatty acids, linoleic acid, alpha-linolenic acid, omega-3 fatty acids

## Abstract

The identification of nutritional patterns associated with the development of type 2 diabetes (T2D) might help lead the way to a more efficient and personalized nutritional intervention. Our study is aimed at evaluating the association between fatty acids (FA) in red blood cell (RBC) membranes, as a quantitative biomarker of regular dietary fat intake, and incident type 2 diabetes in a Spanish population. We included 1032 adult Spaniards (57% women, age 49 ± 15 years, 18% prediabetes), without diabetes at study entry, from the Di@bet.es cohort. Incident diabetes was diagnosed at the end of the study follow-up. The FA percentage in RBC was determined at baseline by gas chromatography. Participants were followed on average 7.5 ± 0.6 years. Lower percentages of linoleic acid (LA), α-linolenic (ALA), and eicosapentaenoic acid (EPA), and higher percentages of docosahexaenoic acid (DHA) in RBC membranes were associated, independently of classical risk factors, with worse glucose metabolism at the end of the study follow-up. In addition, higher percentages of ALA and EPA, and moderate percentages of DHA, were associated with lower risk of diabetes. No significant associations were found for LA and diabetes risk. Dietary patterns rich in vegetables are independently associated with lower risk of both deterioration of glucose regulation and incident diabetes, and should be reinforced for the prevention of diabetes.

## 1. Introduction

Diabetes caused 1.6 million deaths worldwide in 2016 [1], and it is estimated that 578 million people will have diabetes in 2030 and about 700 million by 2045 [2], with 90% of them accounting for type 2 diabetes. In Spain, the prevalence of type 2 diabetes is around 11–14% [3,4] of the population, with an incidence of 12 cases/1000 person-years [5]. Considering that type 2 diabetes is a potentially preventable non-communicable disease [2], the identification of nutritional patterns associated with type 2 diabetes might contribute to an improved and more personalized nutritional intervention, aimed at reducing its incidence.

Prospective studies evaluating the association between diet and type 2 diabetes mostly rely on dietary questionnaires to quantify regular fatty acid (FA) intake. However, self-reported intakes are subject to recall and subjective biases or selective reporting, as well as subjected to deviations according to nutrient databases [6]. Therefore, biological markers might be more accurate and precise in the quantification of FA intake than self-reported questionnaires. The FA composition of red blood cell (RBC) membranes has been widely used as a surrogate objective biomarker of long-term intake of dietary fatty acids, with absent or marginal endogenous synthesis, including essential FA (linoleic and α-linolenic acids) and long-chain omega 3 FA, such as eicosapentaenoic (EPA) and docosahexaenoic (DHA) acids [7]. Therefore, quantifying the FA pattern in RBC membranes might, at least partially, solve the limitations associated with the quantification of FA intake through dietary questionnaires.

Epidemiological studies evaluating the relative content of FA in blood or tissues, as biomarkers of FA intake, and the risk of type 2 diabetes, have shown conflictive or apparently contradictory results [8,9,10,11,12]. These controversies pinpoint that the intake of FA may be important in modulating the risk of developing type 2 diabetes, although the specific contribution of each fatty acid is unresolved. Accordingly, the dietary fatty acid profile, the food matrix and the cooking procedures may be of much more relevance than the intake of a single FA. In this regard, just a few studies have analyzed the association between some FA, as a surrogate marker of specific foods intake, and the incidence of type 2 diabetes [8,9,13]. However, there is no data considering all polyunsaturated FA (PUFA) biomarkers of intake, providing a more reliable measure of the dietary pattern rather than a specific food group consumption. In addition, in the majority of these studies, diabetes diagnosis is based on self-reported or registry data and, thus, no information on the progression to a worse glucose metabolism is provided. Therefore, our study was aimed at evaluating the association between the FA species of RBC membranes, used as a quantitative biomarker of regular dietary intake, and the development of type 2 diabetes or the risk of progression of glycemic metabolism category, in a Spanish population.

## 2. Materials and Methods

### 2.1. Study Subjects

Subjects from this study are part of the Di@bet.es Study cohort who participated in the diabetes incidence study [5]. The Di@bet.es Study is a population-based study aimed at determining the prevalence and incidence of diabetes in a representative sample of Spanish adult individuals. For an extensive characterization of the complete cohort, please refer to references [4,5]. 

Briefly, inclusion criteria for the baseline selection (2008–2010) were being older than 18 years old, and exclusion criteria were chronic serious illness, recent surgery, pregnancy/lactation or disability. Participants without diabetes were invited to another clinical evaluation in 2016–2017. The final Di@bet.es Study incidence cohort included 2408 individuals, 293 (12%) with prediabetes [5]. In the present study we included 1032 subjects, 43% of the Di@bet.es Study incidence cohort. This represents a convenient sample selected according to the following sequence of criteria: (1) availability of frozen blood samples to measure fatty acids, (2) inclusion of all the individuals with baseline prediabetes (*n* = 185), and (3) random selection of a non-prediabetic group at least four times as large as the prediabetic one (*n* = 847, 4.6-fold larger that the prediabetic group). Subjects were followed-up for a mean of 7.5 ± 0.6 years (second visit during 2016–2017). Both at inclusion and follow-up, oral glucose tolerance test (OGTT) for individuals with fasting capillary glucose <7.8 mmol/L (measured by OneTouch^®^ system, Lifescan, Johnson & Johnson, S.A., Madrid, Spain), anthropometric, and biochemical analyses were performed. Glucose was determined by the hexokinase enzymatic method and HbA1c (only at follow-up) by high-performance liquid chromatography (analyzer ADAMS A1C HA-8180V, ARKRAY, Minneapolis, MN, US). In addition, sociodemographic, medical, and dietary data were also recorded at both time points by the same structured questionnaire including closed questions regarding sex, age, educational level (none, basic, high school or college), personal medical history of diabetes, hypertension, or dyslipidemia, family history of diabetes, medications (with a particular focus on diabetes, blood pressure or dyslipidemia treatment), a physical activity (International physical activity questionnaire, IPAQ), and a semiquantitative food frequency consumption questionnaire. The Homeostatic Model Assessment for Insulin Resistance (HOMA-IR) was calculated as (insulin -UI- x glucose -mmol/L-)/22.5, waist to hip ratio was obtained by dividing waist circumference (cm) with hip circumference (cm), and the percentage of body fat was calculated with the *Clínica Universidad de Navarra-Body Adiposity Estimator* (CUN-BAE) equation [14].

All procedures were carried out in accordance with the Declaration of Helsinki, and the study was approved by the Ethics and Clinical Research Committee of the Hospital Regional Universitario de Malaga (Malaga, Spain). All participants provided written informed consent prior to inclusion in the study.

### 2.2. Clinical and Laboratory Analyses

Blood was withdrawn in fasting conditions and frozen at −80 °C for FA analyses. Biochemical measurements were performed with standard procedures. Glucose was determined with the hexokinase enzymatic method. Fasting and 2-h glucose were quantified before and after the administration of 75 g glucose overload (OGTT). Based on these glucose measurements, individuals were classified at baseline and follow-up in different glucose categories following WHO criteria [15]: normal OGTT, isolated fasting glucose (IFG) between 110 and 125 mg/dL, isolated impaired glucose tolerance (IGT) between 140 and 199 mg/dL after 2-h glucose overload, or both (IFG/IGT).

### 2.3. Quantification of Fatty Acids in the Cell Membrane of Erythrocytes

The percentage of FA in RBC membranes was determined at inclusion by gas chromatography, as previously and extensively described [16,17]. Briefly, fatty acid methyl esters extracted from erythrocytes were quantified with an Agilent 7890A Gas Chromatograph (Agilent, Santa Clara, CA, US) equipped with a 30 m × 0.25 mm × 0.25 mm SupraWAX-280 capillary column (Teknokroma, Sant Cugat del Vallès, Barcelona, Spain), an autosampler, and a flame ionization detector. FA were quantified as relative percentage of the total fatty acids identified in each sample. Saturated FA (SFA) were defined as the sum of the percentages of C14:0, C16:0, C18:0, C20:0, C22:0 and C24:0; monounsaturated FA(MUFA) as the sum of C16:1n7, C18:1n9, C20:1n9, and C24:1n9; and PUFA as the sum of the percentages of C18:2n6, C20:2n6, C20:3n6, C20:4n6, C22:4n6, C22:5n6, C18:3n6, C18:3n3, C20:5n3, C22:5n3, and C22:6n3. From the 21 FA quantified, we mainly focused on linoleic acid (derived from nuts and seeds), α-linolenic acid (contained in nuts, soy, vegetables and olive oil), and EPA and DHA (present mostly in fatty fish). Omega 3 index was calculated as the sum of EPA and DHA.

### 2.4. Definition of Outcomes at Follow-Up

The incidence of diabetes was recorded after a mean of 7.5 ± 0.6 years of follow-up. As previously mentioned, at the end of the study follow-up, glucose and HbA1c were determined, and an OGTT for individuals with fasting capillary glucose <7.8 mmol/L was also performed. Diabetes was defined as: hypoglycemic treatment at the final visit, fasting blood glucose ≥126 mg/dL, HbA1c ≥ 6.5%, and/or blood glucose ≥200 mg/dL after 2 h of oral overload. Prediabetes was defined as IFG, or IGT, or both. According to the glycemic status, at the end of the study follow-up, subjects with incident diabetes and overall progressors (from baseline normal glucose metabolism to pre- or diabetes, or from baseline prediabetes to diabetes) were identified.

### 2.5. Statistical Analyses

Statistical analyses were performed using SPSS version 24 (IBM, Armonk, NY, United States). Descriptive data are presented as mean ± SD for continuous variables and frequency or proportions (%), for categorical variables. Normality of variables was assessed with the Shapiro–Wilk test. Baseline differences were assessed by the Chi Square or t tests, for categorical and numerical variables, respectively.

The percentages of FA were standardized by z-scores (mean  =  0, SD  =  1) before analyses to ensure that odd ratios (ORs) and confidence intervals (CI) for incident diabetes were comparable between them. Diabetes incidence was recorded at the end of the study follow-up, and thus, time-to-event information was not available. In consequence, logistic regression models, adjusting for potential confounders and diabetes predictors, were used to estimate associations between each fatty acid or group of interest (Omega 3 index) and diabetes incidence or progression of glucose metabolism category. All analyses were stratified by the node of recruitment. Moreover, three levels of adjustment were considered for regression analyses: (i) adjusted by baseline age and sex; (ii) further adjusted by first-degree family history of diabetes, baseline body mass index (BMI), and baseline glucose metabolism category (normal, IGF, IGT, and both IGF and IGT); (iii) further adjusted by baseline physical activity (low, moderate and high [18]), central obesity (defined as waist-to-hip ratio >1 or 0.85 for men and women, respectively), educational level (defined in 4 categories, from none to superior studies), and hypertension. Nonlinearity in the associations was explored with the use of quintiles. ROC Curve analyses were performed to evaluate the utility of some specific FA and glucose as predictive biomarkers of progression in glucose metabolism category and diabetes. Sensitivity analyses were performed excluding subjects with prediabetes at baseline. A 2-tailed *p* < 0.05 was considered significant.

## 3. Results

### 3.1. Subjects Characteristics

Baseline characteristics of the subjects included in the study are shown in Table 1. The mean age was about 50 years, and 58% of the subjects were females. Obesity was found in 30% of the participants, 18% had prediabetes, and 38% presented first-degree family history of diabetes. In addition, the distribution of the fatty acids biomarkers of dietary intake within the study subjects can be found in Table S1.

### 3.2. Incidence of Diabetes after Follow-Up

From the 1032 participants included in the study, 324 subjects (31.4%) were diagnosed with diabetes or prediabetes during the study period or at the final follow-up visit. From them, 131 developed type 2 diabetes (12.7%), during or at the end of the 7.5 ± 0.6 years of follow-up period (Table 2). 

As shown in Table S2, at baseline, subjects who developed diabetes were older and had higher prevalence of obesity and first-degree family history of diabetes. However, no differences in changes in body weight (both in absolute value -Kg- or percentage) were observed at the end of the study, between subjects who developed diabetes or remained without diabetes (*p* = 0.214 and 0.149, respectively). As expected, those subjects with IFG, IGT or both at baseline, presented higher probability of incident diabetes (OR (95% CI): 1.5 (1.2–1.8), 1.3 (1.1–1.4), and 2.5 (1.5–4.3), respectively) compared to those subjects with normal glucose metabolism at inclusion (*n* = 847, 82.1%).

### 3.3. Association between the Fatty Acid Profile and the Risk of Progression in Glucose Metabolism Category

In general terms, higher proportions of saturated FA, n3 PUFA and a high Omega 3 index, in RBC membranes, were associated with increased risk of progression to a worsened glucose metabolism (Figure S1). A higher baseline percentage in RBC membranes of linoleic and α-linolenic acids, and EPA, was associated with a lower risk of progression of glucose metabolism category (Tables S3 and S4). However, higher DHA content was associated with a higher risk of progression. As shown in Table S1, DHA accounts for about 90% of the Omega 3 index. Consequently, the Omega 3 index was also associated with an increased risk of progression to worse glucose metabolism.

**Figure 1:**
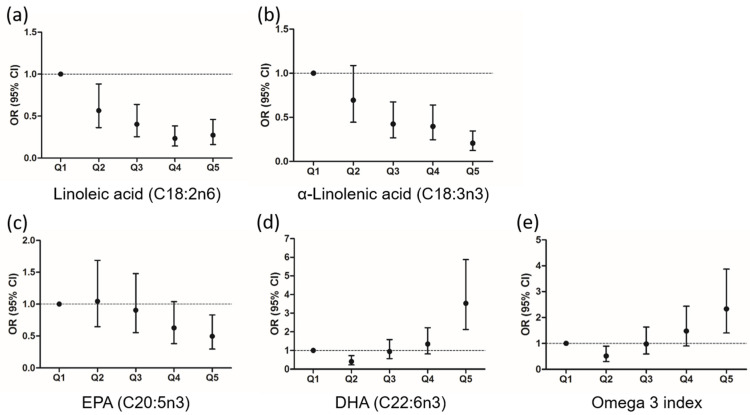
Probability of progression in the glucose metabolism category according to baseline quintiles of fatty acids. Fully adjusted OR, odds ratio; and 95% CI, confidence interval by quintiles (Q) of (**a**) linoleic acid; (**b**) α-linolenic acid; (**c**) EPA; (**d**) DHA; and (**e**) Omega 3 index. Q1 to Q5 are the quintiles of the percentage of each fatty acid in the graphs. EPA indicates eicosapentaenoic acid; DHA, docosahexaenoic acid. Omega 3 index is the sum of EPA and DHA.

To further examine the relationship between RBC FA content and risk of progression to a worse glucose metabolism and explore the existence of non-linear associations, we categorized the sample according to quintiles of each FA content in RBC. As depicted in Figure 1, the probability of progression steadily decreased across quintiles of linoleic acid, α-linolenic acid, and EPA. In contrast, baseline DHA concentrations and the probability of worse glucose metabolism had a nonlinear J-shaped relationship. As illustrated in Figure 1, a low-to-moderate content of DHA (Q2) was associated with a lower probability of progression (OR (95% CI): 0.405 (0.226, 0.726), *p* = 0.002, vs. Q1), while individuals at the highest quintile of DHA content were at higher probability (OR (95% CI): 3.529 (2.119, 5.879), *p* < 0.0001, vs. Q1). The same pattern was observed for omega-3 index.

### 3.4. Association between the Fatty Acid Profile and the Risk of Diabetes

As similarly observed with the progression to a worsened glucose metabolism, higher percentages of saturated FA were associated with increased risk of diabetes, whereas a high proportion of MUFA, n6 PUFA, and a higher ratio of UFA/SFA in RBC membranes, as well as higher D5D, were associated with a lower risk of diabetes (Figure S2).

**Figure 2:**
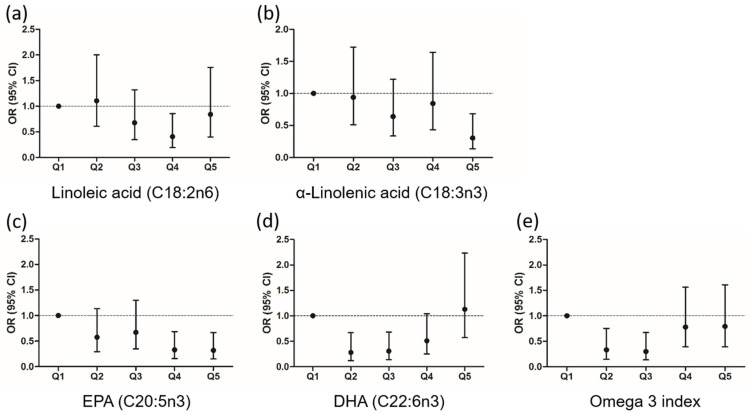
Probability of being diagnosed with diabetes according to baseline quintiles of fatty acids. Fully adjusted OR, odds ratio; and 95% CI, confidence interval by quintiles (Q1) of (**a**) linoleic acid; (**b**) α-linolenic acid; (**c**) EPA; (**d**) DHA; (**e**) Omega 3 index. Q1 to Q5 are the quintiles of the percentage of each fatty acid in the graphs. EPA indicates eicosapentaenoic acid; DHA, docosahexaenoic acid. Omega 3 index is the sum of EPA and DHA.

Higher linoleic acid, α-linolenic acid, and EPA content in RBC were independently associated with a lower risk of diabetes, whereas higher content in DHA was associated with higher probability of incident type 2 diabetes (Tables S5 and S6). However, after adjustment, the association between linoleic acid and diabetes was no longer significant. No associations were found between Omega 3 index and the risk of type 2 diabetes. Again, as depicted in Figure 2, the probability of incident diabetes decreased across quintiles of α-linolenic acid and EPA, showing an inverse linear trend. Conversely, DHA and Omega 3 index showed a J-shaped relationship, with significant risk reductions of type 2 diabetes observed for Q2 and Q3 (OR (95% CI): 0.281 (0.119, 0.667) and 0.308 (0.139, 0.682), for Q2 and Q3, respectively, *p* = 0.004 both), compared with Q1.

### 3.5. Red Blood Cells Fatty Acids as Predictive Biomarkers of Progression in Glucose Metabolism Category and Diabetes

ROC Curve analyses were performed to explore the capacity of RBC FA to predict the progression in glucose metabolism category and diabetes in 7 years. Linoleic acid in percentages below 12.40% in RBC predicted a progression to a worse glucose metabolism and diabetes onset with an area under the curve (AUC) 95% confidence interval (CI) = 0.685 (0.649, 0.721) and 0.632 (0.578, 0.685), for progression and diabetes, respectively (*p* < 0.0001, both). Percentages below 0.13 of α-linolenic acid in RBC were also predictive of progression in glucose metabolism category and diabetes in 7 years (AUC (95% CI) = 0.657 (0.620, 0.693) and 0.610 (0.660, 0.560), respectively, *p* < 0.0001, both). As expected, fasting blood glucose also predicted progression in glucose metabolism category and diabetes at 7 years (AUC (95% CI) = 0.681 (0.646, 0.717) and 0.757 (0.709, 0.810), respectively, *p* < 0.0001, both). 

### 3.6. Analysis of Sensitivity

Sensitivity analyses to avoid potential reverse causality were performed, excluding subjects with prediabetes (*n* = 185, 17.9%) at baseline. From the 847 subjects with normal glucose metabolism at inclusion, 254 (30%) progressed to a worse glucose metabolism category, from whom 61 (7.2%) developed type 2 diabetes during the study follow-up. As shown in Table S7, after multivariable-adjustment, the associations between linoleic acid, α-linolenic acid, EPA, DHA and the Omega 3 Index, with the probability of progression in glucose metabolism status or incident diabetes unchanged, or strengthened, as compared with the analyses including subjects with prediabetes. However, when excluding subjects with prediabetes, the negative association between EPA and type 2 diabetes risk was no longer statistically significant. Therefore, for incident diabetes (Table S8), statistical significance only remained for α-linolenic acid and DHA in fully-adjusted models.

## 4. Discussion

In this subset of a Spanish cohort, representative of the adult population, the most robust finding was that the higher the percentage of α-linolenic acid in RBC membranes, the lower the risk of progression into a worse glucose metabolism and type 2 diabetes incidence. To date, the associations between omega 6 and omega 3 FA and type 2 diabetes risk are inconclusive and controversial. 

We have observed an inverse association between linoleic and α-linolenic acids content in RBC membranes and glucose metabolism category deterioration and incident diabetes. Our findings are in accordance with the EPIC-InterAct Case-Cohort Study [10], in which authors also observed that α-linolenic acid in plasma phospholipids was associated with decreased risk of type 2 diabetes. They also observed an inverse association between linoleic acid and risk of type 2 diabetes, which, in our cohort, only lost significance after multivariable adjustment. Oppositely, in the in EPIC-Potsdam study [19], erythrocyte α-linolenic acid was not associated with type 2 diabetes, although linoleic acid was associated with a lower risk of type 2 diabetes. In the same vein, linoleic acid contained in plasma cholesteryl esters was inversely associated with type 2 diabetes risk in Dutch patients who suffered a myocardial infarction [20], in a pooled analysis from 20 prospective studies from different countries, including measurements of FA in different compartments and comparing the FA profile of different lipid compartments [13], and in two Finnish cohorts [8,21]. It is worth mentioning that in the first Finnish cohort, no association was found for serum α-linolenic acid and the risk of type 2 diabetes [8]. This lack of association for α-linolenic and type 2 diabetes was also observed in a Chinese population [22], in a prospective cohort of middle-aged women [11], and in another pooled analysis from 20 prospective cohorts [9]. However, in a different Chinese cohort [23], linoleic acid was not associated with increased incidence of type 2 diabetes, in accordance with our results. In addition, in The Multi-Ethnic Study of Atherosclerosis [24], linoleic acid contained in plasma phospholipids was inversely associated with type 2 diabetes risk, although no longer significant after stratification by ethnicity. In a very recent meta-analysis [25], linoleic acid in blood or adipose tissue was also inversely associated with type 2 diabetes risk, although with a moderate certainty of evidence. It, therefore, appears that omega 6 and omega 3 FA from plant origin are inversely associated with type 2 diabetes risk, although the strength of the association may depend on the ethnicity studied [26], and inherently, the cultural and geographical dietary pattern, or the lipid compartment evaluated.

Our results are also endorsed by the findings of a recent meta-analysis, in which high intake of vegetable fat, and more specifically α-linolenic acid, was associated with lower risk of type 2 diabetes, whereas marine omega 3 PUFA (EPA and DHA) were associated with a higher risk of type 2 diabetes [27]. Again, in accordance with our results, in a Chinese cohort, erythrocyte membrane EPA was negatively associated with type 2 diabetes and DHA showed the opposite (hazard ratio (95% CI) in quartile 4: 1.52 (0.99, 2.34)), although this association did not reach statistical significance [22]. Recent studies evaluating the effects of marine omega 3 supplementation in major adverse cardiovascular events have shown controversial results. In the REDUCE-IT study, 4 g/day supplementation of EPA compared to mineral oil supplementation resulted in an absolute between-group difference of 4.8% of ischemic events (hazard ratio (95% CI): 0.75 (0.68, 0.83)) [28]. However, in the OMEMI [29] or the STRENGHT [30] trials, such risk reduction was not observed after marine omega 3 (mix of EPA and DHA) supplementation, suggesting that the biological effects of EPA and DHA may be divergent. In the same line, we have observed opposite associations of erythrocytic EPA and DHA and the risk of diabetes. Despite decades of study on the effects of marine omega 3 in type 2 diabetes, there is still a lack of conclusive evidence on the role of these omega 3 in type 2 diabetes onset and progression [31]. The relationship between fatty fish consumption, marine omega 3 and type 2 diabetes seems largely influenced by the geographical region [12], and also by the incorporation rates of EPA and DHA in erythrocytic membranes, which appears to be irregular for DHA and stable for EPA [32]. This phenomenon may explain, at least in part, that despite fish consumed in Spain containing, on average, 2.5–fold more DHA than EPA, DHA in erythrocytic membranes accounts for the 90% of the Omega 3 index. In this setting, single country/region studies, such as ours, appear to be as important as pooled regions studies to decipher this complex relationship. In a meta-analysis of 10 prospective cohorts, EPA and DHA were associated with increased risk of type 2 diabetes [33]. However, in the EPIC-InterAct Case-Cohort Study, no significant associations between erythrocytic EPA or DHA and the risk of type 2 diabetes were found [10], as also observed in the EPIC-Potsdam study [19], and in a Finnish cohort [21]. Oppositely, in a prospective cohort of women aged 55 years at inclusion, the marine omega 3 PUFA EPA, and DHA to a higher extent, were associated with increased risk of type 2 diabetes [11]. Contrarily, in another recent study of 20 pooled cohorts EPA, DHA and DPA were inversely associated with type 2 diabetes incidence [9]. However, and in agreement with our findings, non-linear associations were found between the estimated intake of DHA (and marine omega 3, by extension) and risk of type 2 diabetes, with opposite directions between Asian and Western countries [27,33]. These results suggest that the intensity of the associations between FA and type 2 diabetes may depend on, not only the quality of the diet (FA profile, dietary context and cooking procedures), but also on the quantity. In this context, we have analyzed the OR of single and types of FA previously standardized to mean = 0 and standard deviation = 1, for each of the FA analyzed. In light of our results, and the overall controversies, further studies are urged to determine the threshold amounts of marine omega 3, which may be beneficial or neutral for type 2 diabetes, and even cardiovascular disease, to elucidate the potential differential biological activities of EPA and DHA [34], and nutritional recommendations should embrace this information.

To our knowledge, the epidemiological data associating the fatty acid profile with the progression of glycemic category are scarce. We have observed consistent and stronger associations for the risk of progression in glycemic category than for the risk observed only in those progressing to type 2 diabetes, suggesting that a healthy dietary fat pattern consumption would be effective in maintaining normal glucose metabolism. Therefore, a life-long healthy dietary pattern may be very effective for type 2 diabetes prevention and maintaining a normal glucose metabolism. In fact, our ROC analysis shows that, in addition to traditional risk factors, the quantification of linoleic acid and α-linolenic acid in RBC might be a useful tool to intensify dietary counselling in the clinical setting, in addition to other diabetes prevention strategies, for the prevention of progression of glycemic category.

Overall, our results indicate that, in the Spanish population, a diet rich in vegetable fat, and by inference, low in animal fat, typical features of the Mediterranean diet, may contribute to lowering the risk of progression of glycemic category and type 2 diabetes. The associations between blood/tissue PUFA with the risk of type 2 diabetes might be region-dependent and, thus, largely influenced by the dietary pattern itself. It is noteworthy, that the current body of evidence suggests that dietary counselling aimed at preventing type 2 diabetes may endorse a diet based on the consumption of plant-based products.

Major strengths of this study are the quantification of objective biomarkers of fat intake (averaging a 3-month period parallel to erythrocyte half-life) instead of food frequency questionnaires, the population-based prospective design, and the lengthy follow-up period (>7 years). Moreover, diabetes was accurately diagnosed at the end of the study follow-up, which allowed evaluating the progression to a worse glucose metabolism, crucial to prevent type 2 diabetes onset. However, this study is not exempt of limitations. We were not able to perform Cox regression analyses because diabetes incidence was registered at the end of the study follow-up. The relative contribution of snacks versus fresh vegetables in the linoleic and α-linolenic acids composition of RBC could not be quantified. However, it is very improvable that the negative association between these two fatty acids and the risk of progression to a worse glucose metabolism or type 2 diabetes might be conveyed through the consumption of processed foods, the consumption of which has been associated with increased incidence of type 2 diabetes [35]. The study subjects belong to the Spanish population-based study [5] and, therefore, with limited generalizability to other populations, such as Asian ethnicities [27,33]. We quantified FA only at baseline and, thus, potential changes over time in fat consumption may be overlooked. Nevertheless, dietary habits of the adult population are relatively stable within 7 years, and given the magnitude of the associations, it is unlikely that they might be explained by changes in the dietary habits over time. Finally, given the observational nature of the study design, residual confounding cannot be discarded. 

## 5. Conclusions

The risk of prediabetes and type 2 diabetes is closely linked to the quality and quantity of the diet. Our data indicate that a diet rich in vegetable fat may contribute to delaying the progression to a worse glucose metabolism category and, therefore, to the onset of type 2 diabetes. 

## Figures and Tables

**Table 1:** Baseline characteristics of the subjects included in the study.

	All (*n* = 1032)	Normoglycemia (*n* = 847)	Prediabetes (*n* = 185)	*p*
Female sex	596 (57.8)	488 (57.6)	108 (58.4)	0.849
Age, years	49.41 ± 14.94	48.01 ± 14.94	55.79 ± 13.25	<0.0001
Hypertension	219 (21.2)	153 (18.1)	66 (35.7)	<0.0001
Family history of diabetes	389 (37.7)	312 (36.8)	77 (41.6)	0.004
Dyslipidemia	340 (32.9)	265 (31.3)	75 (40.5)	0.007
Sedentarism	75 (7.5)	52 (6.1)	23 (12.4)	0.002
Glucose metabolism category				<0.0001
Normoglycemia	847 (82.1)	847 (100)	-	
Impaired fasting glucose (IFG)	61 (5.9)	-	61 (33.0)	
Impaired glucose tolerance (IGT)	98 (9.5)	-	98 (53.0)	
IFG and IGT	26 (2.5)	-	26 (14.0)	
Glucose, mmol/L	5.22 ± 0.67	5.09 ± 0.58	5.81 ± 0.71	<0.0001
Insulin, pmol/L	66.60 ± 36.89	61.80 ± 31.74	88.77 ± 49.04	<0.0001
HOMA-IR	2.28 ± 1.44	2.05 ± 1.16	3.34 ± 2.02	<0.0001
Waist-to-hip ratio	0.89 ± 0.09	0.88 ± 0.09	0.93 ± 0.08	<0.0001
Body fat, %	33.09 ± 9.36	32.22 ± 9.41	37.09 ± 8.03	<0.0001
BMI, Kg/m^2^	27.94 ± 4.75	27.33 ± 4.43	30.79 ± 5.12	<0.0001
BMI > 30 Kg/m^2^	306 (29.7)	210 (24.8)	96 (51.9)	<0.0001
Total cholesterol, mg/dL	199.99 ± 40.52	198.52 ± 40.14	206.74 ± 41.67	0.014
cHDL, mg/dL	52.45 ± 13.15	52.80 ± 13.25	50.85 ± 12.59	0.073
cLDL, mg/dL	108.40 ± 30.49	107.15 ± 29.99	114.09 ± 32.15	0.006
Triglycerides, mg/dL	122.90 ± 104.06	118.95 ± 109.38	140.98 ± 72.63	0.010

Results are expressed as mean ± standard deviation or number (percentage) as appropriate. *p* from the comparison between participants with normal glucose metabolism and prediabetes at inclusion. HOMA-IR indicates Homeostatic Model Assessment for Insulin Resistance, and BMI, Body Mass Index.

**Table 2:** Progression in the glucose metabolism category during the follow-up in the subjects included in the study.

*n* = 1032	Baseline	End of the Follow-Up
Diabetes Mellitus (DM)	0 (0)	131 (12.7)
Known DM	0 (0)	70 (6.8)
Unknown DM	0 (0)	61 (5.9)
Glucose metabolism category		
Impaired fasting glucose (IFG)	61 (5.9)	72 (7.0)
Impaired glucose tolerance (IGT)	98 (9.5)	104 (10.1)
IFG and IGT	26 (2.5)	51 (4.9)
Prediabetes	185 (17.9)	227 (22.0)
Progressors	-	324 (31.4)

Results are expressed as number of subjects (percentage of the total population). Progressors are participants who progressed to a worse glucose metabolism category at the end of the study period as compared to the baseline situation.

## Data Availability

The data presented in this study are available on request from the corresponding author. The data are not publicly available due to protection of participants’ personal data.

## Supplementary Materials

The following supporting information can be downloaded at: https://www.mdpi.com/article/10.3390/nu14071368/s1, Figure S1: Probability of progression in the glucose metabolism category according to baseline fatty acid group; Figure S2: Probability of being diagnosed with diabetes according to baseline fatty acid group; Table S1: Red blood cell membrane fatty acid profile in the study population; Table S2: Characteristics at baseline, and changes at the end of the study according to diabetes incidence at the end of the study follow-up.; Table S3: Probability of progression in the glucose metabolism category during the study period according to baseline fatty acids; Table S4: Differences in baseline fatty acid profile according to progression to a worse glucose metabolism at the end of the study follow-up; Table S5: Probability of being diagnosed with diabetes during the study period according to baseline fatty acids; Table S6: Differences in baseline fatty acid profile according to diabetes incidence at the end of the study follow-up; Table S7: Probability of progression in the glucose metabolism category during the study period according to baseline fatty acids excluding subjects with prediabetes at inclusion; and Table S8: Probability of being diagnosed with diabetes during the study period according to baseline fatty acids excluding subjects with prediabetes at inclusion.
