# Supplementary material for: Low Percentage of Vegetable Fat in Red Blood Cells Is Associated with Worse Glucose Metabolism and Incidence of Type 2 Diabetes
Source: Nutrients. 2022 Mar 25;14(7):1368. doi: 10.3390/nu14071368 (PMC9002701; doi:10.3390/nu14071368)
Supplement: Supplementary file 1 [file nutrients-14-01368-s001.zip › nutrients-1650576-Supplementary Material.pdf]

SUPPLEMENTAL MATERIAL FOR Chiva-Blanch G, et al. "Low Percentage of Vegetable Fat in Red Blood Cells Is Associated with Worse Glucose Metabolism and Incidence of Type 2 Diabetes".

Table S1. Red blood cell membrane fatty acid profile in the study population.

Fatty acid	All (n=1032)	Normoglycemia (n=847)	Prediabetes (n= 185)	P^a	P^b
Linoleic acid (C18:2n6)	13.05 (11.51, 14.46)	13.18 (11.67, 14.60)	12.40 (10.90, 13.98)	0.027	0.073
α-Linolenic acid (C18:3n3)	0.15 (0.11, 0.23)	0.15 (0.11, 0.24)	0.13 (0.10, 0.21)	0.230	0.462
EPA (C20:5n3)	0.64 (0.46, 0.92)	0.64 (0.46, 0.93)	0.63 (0.46, 0.87)	0.058	0.206
DHA (C22:6n3)	5.46 (4.57, 6.44)	5.43 (4.51, 6.36)	5.74 (4.73, 6.81)	0.530	0.474
Omega 3 Index	6.19 (5.10, 7.40)	6.13 (5.03, 7.33)	6.49 (5.45, 7.53)	0.943	0.758

Results are expressed as median (quartile 1, quartile 3) of the percentage of each fatty acid relative to the total fatty acids quantified. P^a from the comparison between subjects with normal glucose category and with prediabetes adjusted by age and sex; and P^b further adjusted by body mass index. EPA indicates eicosapentaenoic acid; and DHA, docosahexaenoic acid. Omega 3 index is the sum of EPA and DHA.

Table S2. Characteristics at baseline, and changes at the end of the study according to diabetes incidence at the end of the study follow-up.

	**Non-Diabetes (n=901)**	**Diabetes (n=131)**	***P***
Follow-up, years	7.48 ± 0.59	7.52 ± 0.55	0.372
Females, n (%)	527 (58.5)	69 (52.4)	0.208
Age at baseline, years	48.34 ± 15.05	56.76 ± 11.82	<0.0001
Body weight at baseline, Kg	73.70 ± 14.15	81.3 ± 15.71	<0.0001
Change at the end of the study, Kg	1.24 ± 6.42	0.46 ± 6.61	0.201
Change at the end of the study, %	1.95 ± 8.49	0.74 ± 8.36	0.133
BMI at baseline, Kg/m^2	27.51 ± 4.57	30.95 ± 4.88	<0.0001
Change at the end of the study	0.45 ± 2.41	0.15 ± 2.59	0.223
Obesity at baseline, n (%)	237 (26.3)	69 (52.7)	<0.0001
At the end of the study	272 (30.2)	69 (52.7)	<0.0001
Waist-to-hip ratio at baseline	0.89 ± 0.09	0.94 ± 0.07	<0.0001
Change at the end of the study	0.01 ± 0.07	0.01 ± 0.06	0.838
HOMA-IR at baseline	2.15 ± 1.3	3.19 ± 1.93	<0.0001
Change at the end of the study	0.49 ± 2.48	1.95 ± 10.29	0.053
Family history, first degree, n (%)	319 (35.4)	70 (53.4)	0.001
Glucose metabolism category, n (%)			<0.0001
Impaired fasting glucose (IFG)	37 (4.1)	24 (18.3)	
Impaired glucose tolerance (IGT)	69 (7.7)	29 (22.1)	
IFG and IGT	9 (1.0)	17 (13.0)	
Prediabetes, n (%)	115 (12.8)	70 (53.4)	<0.0001

Results are expressed as mean ± standard deviation or number (percentage) as appropriate. *P* for the comparison between subjects with and without diabetes at the end of the study follow-up (*t* test for unpaired samples in the case of continuous variables, and chi square test for categorical variables). BMI indicates body mass index, and HOMA-IR, Homeostatic Model Assessment of Insulin Resistance.

Table S3. Probability of progression in the glucose metabolism category during the study period according to baseline fatty acids.

**Fatty acid**	**OR (95% CI)**	***P***
Linoleic acid (C18:2n6)		
Model[a]	0.60 (0.51, 0.72)	<0.001
Model[b]	0.61 (0.51, 0.73)	<0.001
Model[c]	0.61 (0.51, 0.73)	<0.001
α-Linolenic acid (C18:3n3)		
Model[a]	0.46 (0.36, 0.59)	<0.001
Model[b]	0.48 (0.37, 0.61)	<0.001
Model[c]	0.49 (0.38, 0.63)	<0.001
EPA (C20:5n3)		
Model[a]	0.71 (0.60, 0.84)	<0.001
Model[b]	0.72 (0.60, 0.86)	<0.001
Model[c]	0.73 (0.61, 0.87)	<0.001
DHA (C22:6n3)		
Model[a]	1.84 (1.57, 2.15)	<0.001
Model[b]	1.85 (1.57, 2.18)	<0.001
Model[c]	1.85 (1.57, 2.18)	<0.001
Omega 3 index		
Model[a]	1.56 (1.33, 1.81)	<0.001
Model[b]	1.57 (1.34, 1.84)	<0.001
Model[c]	1.58 (1.34, 1.86)	<0.001

Progression indicates participants whose glucose metabolism deteriorated during the study follow-up (from baseline normal glucose metabolism to pre- or diabetes, or from baseline prediabetes to diabetes). OR, odds ratio; and 95% CI, confidence interval. Fatty acids were all standardized to mean 0 and standard deviation 1 to allow comparison. IGF indicates impaired fasting glucose; IGT, impaired glucose tolerance. EPA: eicosapentaenoic acid; DHA, docosahexaenoic acid. Omega 3 index is the sum of EPA and DHA. Model[a] adjusted by baseline age and sex; Model[b] further adjustment for first-degree family history of diabetes, BMI, and glucose metabolism category (normal, IGF, IGT, and both IGF and IGT); Model[c] further adjustment for baseline hypertension, physical activity (low, moderate and high), central obesity (defined as waist-to-hip ratio >1 or 0.85 for men and women, respectively), and educational level (defined in 4 categories, from none to superior studies).

Table S4. Differences in baseline fatty acid profile according to progression to a worse glucose metabolism at the end of the study follow-up.

Fatty acid	Non-Progressors (n=708)	Progressors (n=324)	P^a	P^b
Linoleic acid (C18:2n6)	13.45 (12.01, 14.81)	11.92 (10.76, 13.35)	<0.0001	<0.0001
α-Linolenic acid (C18:3n3)	0.16 (0.12, 0.25)	0.12 (0.10, 0.17)	<0.0001	<0.0001
EPA (C20:5n3)	0.65 (0.45, 0.94)	0.63 (0.48, 0.87)	<0.0001	0.001
DHA (C22:6n3)	5.20 (4.41, 6.00)	6.29 (5.28, 7.23)	<0.0001	<0.0001
Omega 3 index	5.89 (4.93, 6.94)	6.98 (5.89, 7.99)	<0.0001	<0.0001

Results are expressed as median (quartile 1, quartile 3) of the percentage of each fatty acid relative to the total fatty acids quantified. P^a from the comparison between progressors and non-progressors at the end of the study follow-up adjusted by age and sex; and P^b further adjusted by body mass index. EPA indicates eicosapentaenoic acid; and DHA, docosahexaenoic acid. Omega 3 index is the sum of EPA and DHA.

Table S5. Probability of being diagnosed with diabetes during the study period according to baseline fatty acids.

Fatty acid	OR (95% CI)	*P*
Linoleic acid (C18:2n6)		
Model[a]	0.80 (0.64, 0.99)	0.040
Model[b]	0.86 (0.68, 1.09)	0.221
Model[c]	0.85 (0.67, 1.09)	0.204
α-Linolenic acid (C18:3n3)		
Model[a]	0.52 (0.36, 0.73)	<0.0001
Model[b]	0.57 (0.39, 0.82)	0.002
Model[c]	0.56 (0.39, 0.82)	0.003
EPA (C20:5n3)		
Model[a]	0.66 (0.52, 0.84)	0.001
Model[b]	0.71 (0.54, 0.92)	0.010
Model[c]	0.72 (0.55, 0.94)	0.016
DHA (C22:6n3)		
Model[a]	1.28 (1.06, 1.54)	0.010
Model[b]	1.26 (1.02, 1.56)	0.034
Model[c]	1.26 (1.02, 1.56)	0.035
Omega 3 index		
Model[a]	1.14 (0.94, 1.38)	0.174
Model[b]	1.14 (0.92, 1.43)	0.231
Model[c]	1.15 (0.92, 1.44)	0.218

OR, odds ratio; and 95% CI, confidence interval. Fatty acids were all standardized to mean 0 and standard deviation 1 to allow comparison. EPA indicates eicosapentaenoic acid; and DHA, docosahexaenoic acid. Omega 3 index is the sum of EPA and DHA. IGF indicates impaired fasting glucose; IGT, impaired glucose tolerance. Model[a] adjusted by baseline age and sex; Model[b] further adjustment for first-degree family history of diabetes, BMI, and glucose metabolism category (normal, IGF, IGT, and both IGF and IGT); Model[c] further adjustment for baseline hypertension, physical activity (low, moderate and high), central obesity (defined as waist-to-hip ratio >1 or 0.85 for men and women, respectively), and educational level (defined in 4 categories, from none to superior studies).

Table S6. Differences in baseline fatty acid profile according to diabetes incidence at the end of the study follow-up.

Fatty acid	Non-Diabetes (n=901)	Diabetes (n=131)	***P*[a]**	***P*[b]**
Linoleic acid (C18:2n6)	13.21 (11.66, 14.59)	11.96 (10.81, 13.36)	0.037	0.105
α-Linolenic acid (C18:3n3)	0.15 (0.11, 0.24)	0.13 (0.10, 0.18)	<0.0001	0.001
EPA (C20:5n3)	0.65 (0.46, 0.94)	0.58 (0.46, 0.75)	0.001	0.004
DHA (C22:6n3)	5.42 (4.54, 6.32)	6.35 (4.77, 7.15)	0.013	0.014
Omega 3 index	6.13 (5.08, 7.27)	6.94 (5.51, 7.94)	0.184	0.158

Results are expressed as median (quartile 1, quartile 3) of the percentage of each fatty acid relative to the total fatty acids quantified. *P*[a] from the comparison between with and without diabetes at the end of the study follow-up adjusted by age and sex; and *P*[b] further adjusted by body mass index. EPA indicates eicosapentaenoic acid; and DHA, docosahexaenoic acid. Omega 3 index is the sum of EPA and DHA.

Table S7. Probability of progression in the glucose metabolism category during the study period according to baseline fatty acids excluding subjects with prediabetes at inclusion.

Fatty acid	OR (95% CI)	*P*
Linoleic acid (C18:2n6)		
Model[a]	0.54 (0.44, 0.66)	<0.001
Model[b]	0.53 (0.43, 0.66)	<0.001
Model[c]	0.54 (0.44, 0.66)	<0.001
α-Linolenic acid (C18:3n3)		
Model[a]	0.43 (0.32, 0.57)	<0.001
Model[b]	0.45 (0.33, 0.60)	<0.001
Model[c]	0.46 (0.35, 0.62)	<0.001
EPA (C20:5n3)		
Model[a]	0.73 (0.60, 0.88)	0.001
Model[b]	0.73 (0.61, 0.89)	0.002
Model[c]	0.74 (0.61, 0.90)	0.003
DHA (C22:6n3)		
Model[a]	2.19 (1.81, 2.65)	<0.001
Model[b]	2.18 (1.80, 2.65)	<0.001
Model[c]	2.23 (1.57, 2.18)	<0.001
Omega 3 index		
Model[a]	1.59 (1.34, 1.90)	<0.001
Model[b]	1.59 (1.33, 1.90)	<0.001
Model[c]	1.63 (1.36, 1.96)	<0.001

Progression indicates participants whose glucose metabolism deteriorated during the study follow-up (from baseline normal glucose metabolism to pre- or diabetes). OR, odds ratio; and 95% CI, confidence interval. Fatty acids were all standardized to mean 0 and standard deviation 1 to allow comparison. EPA indicates eicosapentaenoic acid; and DHA, docosahexaenoic acid. Omega 3 index is the sum of EPA and DHA. Model[a] adjusted by baseline age and sex; Model[b] further adjustment for first-degree family history of diabetes and BMI; Model[c] further adjustment for baseline hypertension, physical activity (low, moderate and high), central obesity (defined as waist-to-hip ratio >1 or 0.85 for men and women, respectively), and educational level (defined in 4 categories, from none to superior studies).

Table S8. Probability of being diagnosed with diabetes during the study period according to baseline fatty acids excluding subjects with prediabetes at inclusion.

Fatty acid	OR (95% CI)	*P*
Linoleic acid (C18:2n6)		
Model[a]	0.85 (0.62, 1.16)	0.316
Model[b]	0.87 (0.63, 1.20)	0.399
Model[c]	0.85 (0.61, 1.19)	0.347
α-Linolenic acid (C18:3n3)		
Model[a]	0.45 (0.26, 0.78)	0.004
Model[b]	0.48 (0.28, 0.82)	0.007
Model[c]	0.48 (0.27, 0.84)	0.009
EPA (C20:5n3)		
Model[a]	0.73 (0.53, 1.02)	0.067
Model[b]	0.75 (0.54, 1.05)	0.095
Model[c]	0.78 (0.55, 1.09)	0.145
DHA (C22:6n3)		
Model[a]	1.44 (1.10, 1.88)	0.008
Model[b]	1.41 (1.07, 1.86)	0.014
Model[c]	1.48 (1.12, 1.96)	0.006
Omega 3 index		
Model[a]	1.28 (0.96, 1.69)	0.087
Model[b]	1.27 (0.95, 1.69)	0.101
Model[c]	1.33 (0.99, 1.78)	0.055

OR, odds ratio; and 95% CI, confidence interval. Fatty acids were all standardized to mean 0 and standard deviation 1 to allow comparison. EPA indicates eicosapentaenoic acid; and DHA, docosahexaenoic acid. Omega 3 index is the sum of EPA and DHA. Model[a] adjusted by baseline age and sex; Model[b] further adjustment for first-degree family history of diabetes and BMI; Model[c] further adjustment for baseline hypertension, physical activity (low, moderate and high), central obesity (defined as waist-to-hip ratio >1 or 0.85 for men and women, respectively), and educational level (defined in 4 categories, from none to superior studies).

Figure S1. Probability of progression in the glucose metabolism category according to baseline fatty acid group.

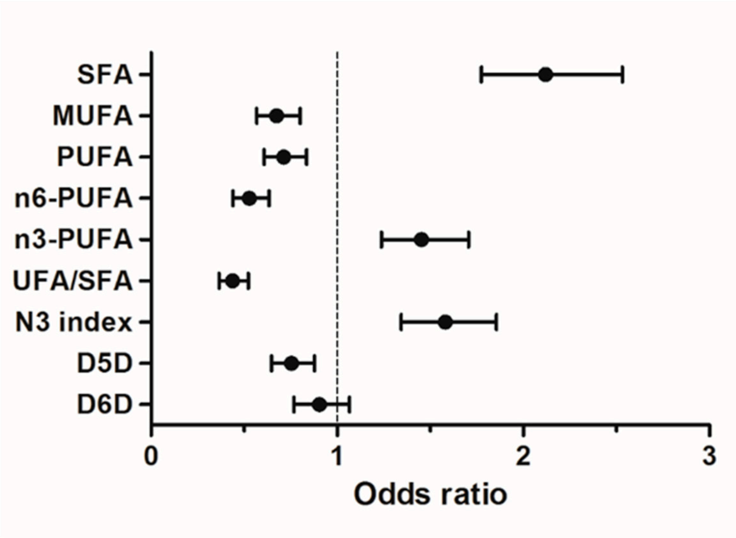

OR (95% CI)	*P*
2.12 (1.78, 2.53)	<0.0001
0.67 (0.57, 0.8)	<0.0001
0.71 (0.61, 0.84)	<0.0001
0.53 (0.44, 0.63)	<0.0001
1.45 (1.24, 1.7)	<0.0001
0.44 (0.37, 0.52)	<0.0001
1.58 (1.34, 1.86)	<0.0001
0.75 (0.65, 0.88)	<0.0001
0.9 (0.77, 1.07)	0.233

OR, odds ratio; and 95% CI, confidence interval. P-values for fully adjusted models (Model 3 at table 3-4) are shown. Fatty acids were all standardized to mean 0 and standard deviation 1 to allow comparison. All analyses were stratified by node of recruitment.

SFA indicates saturated fatty acids; MUFA, monounsaturated fatty acids; PUFA, polyunsaturated fatty acids; and UFA, unsaturated fatty acids (the sum of MUFA and PUFA). n3 Index is the sum of EPA and DHA. D5D is the ratio between arachidonic acid and eicosaenoic acid, and D6D is the ratio between γ-Linolenic acid and linolenic acid, both as indirect measurements of the desaturation of UFA.

Figure S2. Probability of being diagnosed with diabetes according to baseline fatty acid group.

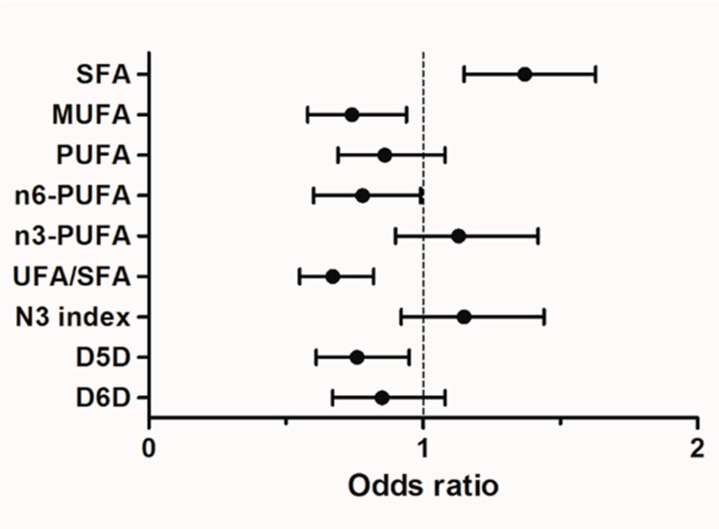

OR (95% CI)	*P*
1.37 (1.15, 1.63)	<0.0001
0.74 (0.58, 0.94)	0.014
0.86 (0.69, 1.08)	0.200
0.78 (0.60, 0.99)	0.048
1.13 (0.90, 1.42)	0.277
0.67 (0.55, 0.82)	<0.0001
1.15 (0.92, 1.44)	0.218
0.76 (0.61, 0.95)	0.016
0.85 (0.67, 1.08)	0.175

OR, odds ratio; and 95% CI, confidence interval. P-values for fully adjusted models (Model 3 at table 3-4) are shown. Fatty acids were all standardized to mean 0 and standard deviation 1 to allow comparison. All analyses were stratified by node of recruitment.

SFA indicates saturated fatty acids; MUFA, monounsaturated fatty acids; PUFA, polyunsaturated fatty acids; and UFA, unsaturated fatty acids (the sum of MUFA and PUFA). n3 Index is the sum of EPA and DHA. D5D is the ratio between arachidonic acid and eicosaenoic acid, and D6D is the ratio between γ-Linolenic acid and linolenic acid, both as indirect measurements of the desaturation of UFA.
